# Supplementary material for: MetaRibo-Seq measures translation in microbiomes
Source: Nat Commun. 2020 Jun 29;11:3268. doi: 10.1038/s41467-020-17081-z (PMC7324362; doi:10.1038/s41467-020-17081-z)
Supplement: Supplementary file 10 — Supplementary Data 7 [file 41467_2020_17081_MOESM10_ESM.zip › File2/Confidence_High_Taxonomy/260026_out.krona.html]

Javascript must be enabled to view this page.

members
magnitude
magnitudeUnassigned
count
unassigned
taxon
rank

260026\_out

290

superkingdom
2
290

290
976
phylum

290
class
200643

order
171549
290

1
family
171552

genus
838
1

1
species
159272

SRS049959\_contig\_number\_40983

287
815
family

287
24

SRS014287\_contig\_number\_590SRS015190\_contig\_number\_contig-100\_782.101715SRS017433\_contig\_number\_contig-100\_3402.97740SRS019068\_contig\_number\_58038SRS019601\_contig\_number\_19842SRS019910\_contig\_number\_19421SRS043411\_contig\_number\_contig-100\_774.107152SRS045645\_contig\_number\_20111SRS045739\_contig\_number\_4530SRS052697\_contig\_number\_40090SRS054905\_contig\_number\_contig-100\_1374.139381SRS055966\_contig\_number\_869SRS063127\_contig\_number\_contig-100\_1222.33270SRS074670\_contig\_number\_contig-100\_1171.35550SRS077231\_contig\_number\_21855SRS077849\_contig\_number\_24295SRS100021\_contig\_number\_8768SRS101376\_contig\_number\_27875SRS1041147\_contig\_number\_1214SRS142923\_contig\_number\_contig-100\_1390.133532SRS143722\_contig\_number\_10515SRS147039\_contig\_number\_1481SRS893288\_contig\_number\_contig-100\_150.29108SRS893327\_contig\_number\_1985
816
genus

2

SRS045244\_contig\_number\_6580SRS062427\_contig\_number\_7733
species
29523

225
821
species

SRS011134\_contig\_number\_33630SRS011239\_contig\_number\_10627SRS011405\_contig\_number\_10570SRS012902\_contig\_number\_514SRS012969\_contig\_number\_25011SRS013382\_contig\_number\_613SRS013521\_contig\_number\_11569SRS013965\_contig\_number\_contig-100\_537.204356SRS014313\_contig\_number\_4520SRS014412\_contig\_number\_13841SRS014534\_contig\_number\_871SRS014683\_contig\_number\_3209SRS014736\_contig\_number\_3580SRS014855\_contig\_number\_1158SRS014948\_contig\_number\_17436SRS014979\_contig\_number\_29928SRS015217\_contig\_number\_25346SRS015369\_contig\_number\_7256SRS015431\_contig\_number\_27755SRS015486\_contig\_number\_283SRS015578\_contig\_number\_33610SRS015663\_contig\_number\_12670SRS015694\_contig\_number\_14001SRS015782\_contig\_number\_27926SRS015854\_contig\_number\_8108SRS015960\_contig\_number\_19300SRS016132\_contig\_number\_758SRS016203\_contig\_number\_contig-100\_552.82905SRS016495\_contig\_number\_contig-100\_2002.115212SRS016629\_contig\_number\_contig-100\_711.35113SRS016753\_contig\_number\_1713SRS016897\_contig\_number\_contig-100\_740.22309SRS016989\_contig\_number\_11051SRS017103\_contig\_number\_24957SRS017307\_contig\_number\_29007SRS017375\_contig\_number\_91SRS017701\_contig\_number\_18269SRS017859\_contig\_number\_2047SRS018351\_contig\_number\_15556SRS018427\_contig\_number\_24965SRS018623\_contig\_number\_13003SRS018817\_contig\_number\_contig-100\_1194.147715SRS018836\_contig\_number\_26664SRS018888\_contig\_number\_4318SRS019030\_contig\_number\_11220SRS019267\_contig\_number\_6877SRS019381\_contig\_number\_16285SRS019397\_contig\_number\_9855SRS019445\_contig\_number\_10779SRS019496\_contig\_number\_4450SRS019693\_contig\_number\_7260SRS019808\_contig\_number\_17610SRS020328\_contig\_number\_21400SRS020508\_contig\_number\_contig-100\_143.21132SRS020622\_contig\_number\_2572SRS021948\_contig\_number\_11727SRS022071\_contig\_number\_25729SRS022713\_contig\_number\_585SRS023829\_contig\_number\_70SRS023971\_contig\_number\_2163SRS024009\_contig\_number\_4246SRS024625\_contig\_number\_10075SRS042284\_contig\_number\_4613SRS043667\_contig\_number\_3164SRS044535\_contig\_number\_22174SRS046068\_contig\_number\_1088SRS046369\_contig\_number\_10399SRS046502\_contig\_number\_11194SRS046810\_contig\_number\_contig-100\_35.36SRS047044\_contig\_number\_16784SRS047433\_contig\_number\_21548SRS049446\_contig\_number\_3066SRS049712\_contig\_number\_142SRS049773\_contig\_number\_34533SRS049995\_contig\_number\_30227SRS050026\_contig\_number\_contig-100\_3047.69705SRS050422\_contig\_number\_26754SRS050520\_contig\_number\_263SRS050752\_contig\_number\_20635SRS050801\_contig\_number\_4026SRS050941\_contig\_number\_496SRS050974\_contig\_number\_1358SRS050998\_contig\_number\_16531SRS051031\_contig\_number\_25066SRS051882\_contig\_number\_1312SRS052576\_contig\_number\_contig-100\_116.15625SRS053087\_contig\_number\_1372SRS053214\_contig\_number\_4960SRS053335\_contig\_number\_16427SRS053398\_contig\_number\_18645SRS053649\_contig\_number\_17103SRS054059\_contig\_number\_4860SRS055017\_contig\_number\_14860SRS056273\_contig\_number\_23191SRS056537\_contig\_number\_10215SRS057478\_contig\_number\_3632SRS057480\_contig\_number\_9706SRS058723\_contig\_number\_1845SRS062701\_contig\_number\_5209SRS063190\_contig\_number\_contig-100\_420.84797SRS063370\_contig\_number\_2384SRS063489\_contig\_number\_2862SRS063518\_contig\_number\_30823SRS063813\_contig\_number\_5844SRS063928\_contig\_number\_106SRS064973\_contig\_number\_31SRS065176\_contig\_number\_3500SRS065397\_contig\_number\_13338SRS074586\_contig\_number\_5133SRS074964\_contig\_number\_contig-100\_3240.51983SRS075398\_contig\_number\_600SRS075716\_contig\_number\_contig-100\_651.44124SRS075821\_contig\_number\_16048SRS075878\_contig\_number\_18518SRS075984\_contig\_number\_24570SRS076756\_contig\_number\_9984SRS076976\_contig\_number\_17284SRS077194\_contig\_number\_26268SRS077294\_contig\_number\_18365SRS077335\_contig\_number\_2214SRS077502\_contig\_number\_contig-100\_823.113687SRS077552\_contig\_number\_646SRS077589\_contig\_number\_2986SRS077641\_contig\_number\_12603SRS077730\_contig\_number\_11736SRS077991\_contig\_number\_contig-100\_102.24009SRS078242\_contig\_number\_21601SRS097958\_contig\_number\_24163SRS098073\_contig\_number\_18059SRS098571\_contig\_number\_30701SRS098644\_contig\_number\_23386SRS098827\_contig\_number\_1784SRS098881\_contig\_number\_21587SRS100907\_contig\_number\_contig-100\_528.62970SRS101433\_contig\_number\_8145SRS103987\_contig\_number\_1785SRS104036\_contig\_number\_contig-100\_660.59160SRS104084\_contig\_number\_4555SRS1041031\_contig\_number\_29124SRS1041036\_contig\_number\_1243SRS1041090\_contig\_number\_5983SRS1041091\_contig\_number\_22346SRS1041092\_contig\_number\_2368SRS1041095\_contig\_number\_1106SRS1041116\_contig\_number\_6013SRS1041118\_contig\_number\_12439SRS1041129\_contig\_number\_4806SRS1041133\_contig\_number\_22818SRS1041134\_contig\_number\_9638SRS1041138\_contig\_number\_6361SRS1041139\_contig\_number\_1592SRS1041140\_contig\_number\_21534SRS1041141\_contig\_number\_9072SRS1041142\_contig\_number\_8505SRS1041143\_contig\_number\_6887SRS1041157\_contig\_number\_693SRS1041164\_contig\_number\_358SRS104327\_contig\_number\_802SRS104400\_contig\_number\_25603SRS104485\_contig\_number\_70SRS104693\_contig\_number\_8355SRS104912\_contig\_number\_17236SRS105082\_contig\_number\_18710SRS1054928\_contig\_number\_6531SRS1055022\_contig\_number\_contig-100\_1030.99461SRS1055038\_contig\_number\_1599SRS1055049\_contig\_number\_10980SRS1055056\_contig\_number\_13940SRS1055076\_contig\_number\_contig-100\_1589.90878SRS1055083\_contig\_number\_contig-100\_891.58940SRS1055095\_contig\_number\_1072SRS142503\_contig\_number\_18713SRS142599\_contig\_number\_359SRS142980\_contig\_number\_1643SRS143070\_contig\_number\_455SRS143085\_contig\_number\_1376SRS143148\_contig\_number\_19863SRS143342\_contig\_number\_9552SRS143372\_contig\_number\_6764SRS143417\_contig\_number\_5089SRS143780\_contig\_number\_12615SRS143876\_contig\_number\_14243SRS143895\_contig\_number\_36549SRS143991\_contig\_number\_23053SRS144362\_contig\_number\_2467SRS144506\_contig\_number\_29938SRS144537\_contig\_number\_44284SRS144753\_contig\_number\_11109SRS145308\_contig\_number\_674SRS146764\_contig\_number\_29073SRS146813\_contig\_number\_18398SRS147022\_contig\_number\_1519SRS147271\_contig\_number\_32449SRS147346\_contig\_number\_2825SRS147557\_contig\_number\_31656SRS147614\_contig\_number\_8525SRS147766\_contig\_number\_6634SRS147977\_contig\_number\_9918SRS148196\_contig\_number\_28173SRS148253\_contig\_number\_2692SRS148424\_contig\_number\_22089SRS148784\_contig\_number\_20647SRS148817\_contig\_number\_contig-100\_1410.85856SRS148970\_contig\_number\_407SRS149075\_contig\_number\_contig-100\_352.102488SRS149181\_contig\_number\_10985SRS149244\_contig\_number\_9868SRS150029\_contig\_number\_19737SRS892882\_contig\_number\_16292SRS893170\_contig\_number\_1695SRS893172\_contig\_number\_10603SRS893187\_contig\_number\_8260SRS893252\_contig\_number\_19793SRS893256\_contig\_number\_contig-100\_4388.28197SRS893259\_contig\_number\_4902SRS893279\_contig\_number\_6064SRS893295\_contig\_number\_12333SRS893341\_contig\_number\_1220SRS893358\_contig\_number\_11982SRS893366\_contig\_number\_10834SRS893369\_contig\_number\_10757SRS893373\_contig\_number\_contig-100\_622.139721SRS893380\_contig\_number\_contig-100\_1373.32636SRS893382\_contig\_number\_1445SRS893383\_contig\_number\_8517

species
310298

SRS014459\_contig\_number\_2262
1


SRS013158\_contig\_number\_contig-100\_345.36562SRS023914\_contig\_number\_23634SRS051610\_contig\_number\_8133SRS1041144\_contig\_number\_6873SRS149325\_contig\_number\_10902
species
371601
5

1

SRS045004\_contig\_number\_318
species
204516


SRS063040\_contig\_number\_21186
1638779
species
1


SRS013687\_contig\_number\_23585SRS017745\_contig\_number\_2526SRS019178\_contig\_number\_3217SRS021484\_contig\_number\_2709SRS077454\_contig\_number\_9778SRS144714\_contig\_number\_11500SRS147425\_contig\_number\_8711
species
46506
7

species
1262750

SRS1041130\_contig\_number\_1884
1

1
47678
species

SRS893285\_contig\_number\_141


SRS058268\_contig\_number\_contig-100\_495.496
species
469593
1

3

SRS015065\_contig\_number\_12817SRS054352\_contig\_number\_18826SRS075773\_contig\_number\_26492
species
246787

28116
species

SRS015794\_contig\_number\_contig-100\_1324.83423SRS142542\_contig\_number\_12917SRS893378\_contig\_number\_7668
3

species
2292935

SRS053356\_contig\_number\_45359
1

2

SRS076876\_contig\_number\_199SRS147377\_contig\_number\_1002
species
820


SRS016095\_contig\_number\_12352SRS019968\_contig\_number\_10990SRS024075\_contig\_number\_698SRS042966\_contig\_number\_1584SRS062654\_contig\_number\_contig-100\_1288.104755SRS104975\_contig\_number\_20266SRS893253\_contig\_number\_1009
818
species
7

1

SRS971275\_contig\_number\_30560
2292944
species

310297
species

SRS078665\_contig\_number\_1897
1

2
family
2005525

2
375288
genus

2
species
46503

SRS016056\_contig\_number\_625SRS017247\_contig\_number\_8427
